# Case report: Prazosin augmentation for treating comorbid treatment-resistant depression and chronic post-traumatic stress disorder

**DOI:** 10.3389/fpsyt.2022.803220

**Published:** 2022-07-22

**Authors:** Ping Guo, Yu Fang, Ming Feng, Xudong Zhao, Shikai Wang, Mincai Qian, Juanjuan Huang, Huanxin Chen

**Affiliations:** ^1^Department of Psychiatry, Huzhou Third Municipal Hospital, The Affiliated Hospital of Huzhou University, Huzhou, China; ^2^Department of Anesthesiology, Huzhou Third Municipal Hospital, The Affiliated Hospital of Huzhou University, Huzhou, China

**Keywords:** Prazosin, post-traumatic stress disorder, treatment-resistant depression, cognitive function, sleep disturbance, comorbidity

## Abstract

Psychological trauma in childhood can lead to post-traumatic disorder (PTSD) with protracted comorbid depression, which responds poorly to conventional antidepressants. Previous studies have shown that prazosin, an α1-adrenergic receptor antagonist, can help eliminate nightmares and improve sleep quality and suicidal ideation in PTSD patients. This case report presents that prazosin had a rapid antidepressant effect in a female adolescent PTSD patient with treatment-resistant depression (TRD). Prazosin improved not only depression symptoms but also sleep quality, suicidal ideation, and cognitive function. Prazosin was well tolerated without obvious adverse effects. Our preliminary study suggests that further clinical trials are needed to determine the efficacy and safety of prazosin in treating PTSD patients with comorbid TRD.

## Introduction

Physical and psychological trauma in childhood is a common precipitating and perpetuating factor for post-traumatic stress disorder (PTSD). Depression is the most common comorbidity of PTSD and often responds poorly to available antidepressants (1–3). Millions of people have post-traumatic stress disorder (PTSD) with comorbid depression (TRD) (3). Selective serotonin reuptake inhibitors (SSRIs) are the first-line pharmacotherapy for treating PTSD (4, 5). However, SSRIs are largely ineffective for the core PTSD symptoms, such as insomnia and nightmares. More than half of patients with PTSD do not achieve remission after treatments with antidepressants and psychotherapy (6), or after repeated applications of the rapid antidepressant, ketamine (7, 8).

Many clinical studies have shown that prazosin, an α1-adrenergic receptor antagonist, significantly improves nightmares in PTSD (9). However, a randomized controlled trial also showed that prazosin was ineffective in alleviating sleep disturbance in PTSD veterans (10). Therefore, more clinical studies are needed to validate the efficacy of prazosin on PTSD. Whether adding prazosin can improve the depressive symptoms in PTSD with comorbid TRD requires further investigation (11).

We here present a case of a female adolescent PTSD patient with comorbid TRD who was previously treated with a wide range of antidepressants, antipsychotics, and electroconvulsive therapy (ECT) but without achieving remission. During this admission, the patient was given low-dose prazosin as an augmenting treatment and showed remarkable improvements in PTSD and TRD symptoms.

## Case report

Miss A, a 16-year-old female with a history of sexual abuse in childhood, has been hospitalized multiple times in the past two years with the diagnosis of depression and PTSD. Patient reported as a victim of sexual assault by a male neighbor when she was 8 years older. She began to suffer from depression, anxiety, and sleep problem 2 years ago. Since then, she has been hospitalized 6 times with major complaints of depression and anxiety. She also complained of sleep disturbance, nightmares, irritation, and memory loss. She often re-experienced the scenario of childhood sexual assault. She thought of killing herself and felt extremely disgusted with adult men. To manage PTSD and depression, she first was on sertraline (100 mg/day) and was late on venlafaxine XR, but with little improvement. Her anxiety was managed with diazepam (10 mg/day) and lorazepam (0.5 mg qn). One year ago, her intimate friend committed suicide from a drug overdose, and she was blamed and threatened by her friend's parents and relatives, and her symptoms worsened. She was augmented with olanzapine (2.5 mg after lunch and 5 mg at bedtime) and co-treated with four MECT and Venlafaxine XR (150 mg/d) but did not get complete remission.

Before this admission, the patient broke up with her partner and attempted to commit suicide multiple times. The patient was hospitalized with the diagnosis of PTSD with comorbid TRD. TRD was diagnosed according to DSM-5 and the failure with two successive antidepressants with adequate dose and duration. Physical exams and image studies were not remarkable. The patient was put on venlafaxine XR (up to 225 mg/day), bupropion XR (up to 300 mg/d), olanzapine (up to 10 mg/day) for 3 weeks, and also received eight sessions of modified electroconvulsive therapy (MECT), but achieved no significant improvement. After informed consent, the patient was first put on prazosin at a low dosage of 0.25 mg at bedtime for 3 days with blood pressure monitored. The dose was then increased to 0.5 mg/day on day 4 and 1 mg/day on day 14 (Table 1). The patient reported no side effects and no significant change in blood pressure. The patient completed scales for depression, anxiety, PTSD, and cognitive functions before and after starting prazosin (Table 2). PTSD symptoms, depressive and cognitive symptoms improved on day 3, and continued to improve after 1, 2, and 4 weeks of treatment. The patient was discharged 4 weeks after the admission and has been followed up. The patient has been on the same medications since discharge. The patient has been doing well and not been hospitalized for mood issues and not reported side effects. She is able to continue her education. Her parents have been satisfied with the improvements. The patient consented to the publication of this case report.

**Table 1:** Timelines of patient events and medications in current admission and follow-ups.

**Date**	**Event**	**Medication/s**	**Comment**
29/Oct-02/Nov/2020	ADMISSION (the 7^th^ time)	Venl 225 mg/d + Ola 7.5 mg qn + Diaz 10 mg ivgtt/d*5 days + MECT(M-W-F)*2 sessions	Broke up with her partner and indulged in video games and tried to kill herself
03-08 /Nov/2020		Venl 225 mg/d + Ola 10 mg qn + Bupr 150 mg/d + Diaz 10 mg ivgtt/d*5 days + MECT(M-W-F)*2 sessions	Bupr augmentation for TRD and reducing the appetite and weight gain caused by Ola
09-18 /Nov/2020		Venl 225 mg/d + Ola 10 mg qn + Bupr 300 mg/d + MECT(M-W-F)*4 sessions	Cognitive deficit after MECT; no improvement in sleep
19/Nov/2020		Venl 225 mg/d+ Ola 10 mg qn +Bupr 300 mg/d+ Lora 1 mg qn + prazosin 0.25 mg qn	Add low-dose prazosin for symptoms of PTSD, especially nightmares
20-22 /Nov/2020		Venl 225 mg/d + Ola 7.5 mg qn +Bupr 300 mg/d+ Lora 1 mg qn + prazosin 0.25 mg qn	Down-titration Ola because of excessive sleep
23-25 /Nov/2020		Venl 225 mg/d+ Ola 7.5 mg qn + Bupr 300 mg/d+Lora 1 mg qn + prazosin 0.5 mg qn	Up-titration prazosin, and nightmare and depression improved
26/Nov/2020	DISCHARGE	Maintain the medications for a month	
26/Dec/2020 -26/Jul/2021	Follow-up as an outpatient	Venl 225 mg/d + Ola 5 mg qn + Bupr 150 mg/d + prazosin 1 mg qn	Ola and Bupr Down-titration, prazosin Up-titration and Lora discontinuation. Her education continued without symptoms of PTSD or TRD relapse
27/Jul/2021 -up to now	Follow-up as an outpatient	Venl 150 mg/d + Ola 2.5 mg qn + Bupr 150 mg/d + prazosin 1 mg qn	Venl and Ola Down-titration. Her social function is good and she feels great about herself.

*Sert, sertraline; Venl, venlafaxine XR; Bupr, bupropion; Ola, olanzapine; Que, quetiapine; Diaz, diazepam; Lora, lorazepam; PTSD, post-traumatic stress disorder; MDD, major depressive disorder; TRD, treatment-resistant depression; MECT, modified electroconvulsive therapy; M-W-F, Monday-Wednesday-Friday; SAE, severe adverse event*.

**Table 2:** Effects of prazosin on heart rate, blood pressure, and emotional and cognitive symptoms of patient.

	**Baseline**	**Day 3**	**Day 7**	**Day 14**	**Day 28**
HR	94	90	86	84	80
BP	124/84	120/80	110/74	110/78	105/75
HAMD_−17_	24	18	10	4	2
HAMA	15	12	8	4	3
CAPS-5	42	28	20	14	12
DSST	45	48	52	53	66
TAT-A(s)	31	24	19	17	12
TAT-B(s)	55	52	49	40	37

*HR, heart rate; BP, blood pressure; HAMD, Hamilton depression rating; HAMA, Hamilton anxiety scale; CAPS-5, the Clinician-Administered PTSD Scale for DSM-5; DSST, Digit Symbol Substitution Test; TMT, Trail-Making Test*.

## Discussion

Depression is a common comorbid complication in PTSD. Like that for depression, selective serotonin reuptake inhibitors (SSRIs) are the first-line drugs for the treatment of PTSD. However, around a half percent of patients respond poorly to the treatment (4, 5). Prazosin was found to improve nightmares in PTSD and has been extensively studied in clinical trials (6, 9). In this case report, we used prazosin in addition to SSRIs to treat PTSD with comorbid TRD. We observed that the addition of prazosin in the treatment regimen gave a rapid onset of antidepressant effect and improved sleep quality, nightmares, cognitive functions, and suicidal ideation.

Previous work mainly investigated the effect of prazosin on PTSD symptoms, especially nightmares (12, 13); less attention has been paid to its possible benefit on depressive symptoms and anxiety. This female adolescent patient suffered from depression and anxiety besides PTSD symptoms. Her depression and PTSD symptoms did not respond to two SSRIs and many sessions of ECT. Adding prazosin to her treatment regimen remarkably improved not only PTSD symptoms but also depressive symptoms and cognitive impairments. Several possible reasons could explain the marked improvement of TRD in the patient by adding prazosin. Prazosin improved nightmares and sleep quality, which might lead to the alleviation of depressive symptoms and anxiety. It is also possible that prazosin itself has an antidepressant action. Preclinical studies reported that α1 antagonists such as prazosin and benoxathian exhibited antidepressant-like activity in the forced swim test and tail suspension test in rodents (14–16) although more experiments are required to support the findings. Another possibility is that prazosin may work together with SSRIs to give more potent antidepressant effects than SSRIs alone. It is worthwhile to conduct further studies to explore this possibility.

We used low doses of prazosin (0.25 to 1 mg daily), which gave marked improvements in both PTSD and depression symptoms without adverse effects. Previous studies showed that prazosin might improve nightmares in children and adolescents with PTSD with rare adverse events, and there was no significant change in blood pressure with the initiation of prazosin (17–19). The dose of prazosin used in previous studies was 1 to 16 mg daily. In adults, the dose of prazosin was up to 45 mg daily (20). The findings in our case report are consistent with previous work on adolescent patients. It was also reported that prazosin was ineffective in alleviating sleep disturbance in PTSD veterans (10). The different findings may imply that prazosin's effect may vary depending on individuals. Age, gender, and comorbidities may be important factors affecting the efficacy of prazosin. Since our current study is a case report, it provides very little information about the roles of these factors in the treatment of PTSD and its comorbid depression and anxiety. Studies with a larger sample size are needed to clarify the roles of these factors.

## Conclusion

Combining low-dose prazosin and SSRIs may effectively improve PTSD symptoms and the comorbid TRD. The use of low-dose prazosin is also safe and well tolerable. However, large-scale clinical studies are warranted to study the findings in this case further.

## Data availability statement

The raw data supporting the conclusions of this article will be made available by the authors, without undue reservation.

## Ethics statement

Ethical approval was obtained from the 3rd Hospital in Huzhou Municipal, Zhejiang, China (Approval No.: AF-39). Written informed consent to participate in this study was provided by the participants' legal guardian/next of kin. Written informed consent was obtained from the individual(s), and minor(s)' legal guardian/next of kin, for the publication of any potentially identifiable images or data included in this article.

## Author contributions

PG: funding acquisition and writing-original draft. YF and XZ: investigation and software. MF: data curation, investigation, and software. MQ: project administration, resources, supervision, and visualization. JH: validation and visualization. SW: conceptualization, methodology, and writing-review & editing. HC: organizing this research and writing and revising this manuscript. All authors contributed to the article and approved the submitted version.

## Funding

This work was supported by the Project of Health Department in Zhejiang Province (2019KY681), Huzhou Municipal Science and Tech Commission (No. 2018GYB16). The funding body had no role in the design of the study and collection, analysis and interpretation of data and in writing the manuscript.

## Conflict of interest

The authors declare that the research was conducted in the absence of any commercial or financial relationships that could be construed as a potential conflict of interest.

## Publisher's note

All claims expressed in this article are solely those of the authors and do not necessarily represent those of their affiliated organizations, or those of the publisher, the editors and the reviewers. Any product that may be evaluated in this article, or claim that may be made by its manufacturer, is not guaranteed or endorsed by the publisher.
